# A Highly Sensitive Assay for Monitoring the Secretory Pathway and ER Stress

**DOI:** 10.1371/journal.pone.0000571

**Published:** 2007-06-27

**Authors:** Christian E. Badr, Jeffrey W. Hewett, Xandra O. Breakefield, Bakhos A. Tannous

**Affiliations:** 1 Molecular Neurogenetics Unit, Department of Neurology, Massachusetts General Hospital, Boston, Massachusetts, United States of America; 2 Center for Molecular Imaging Research, Department of Radiology, Massachusetts General Hospital, Boston, Massachusetts, United States of America; 3 Program in Neuroscience, Harvard Medical School, Boston, Massachusetts, United States of America; Newcastle University, United Kingdom

## Abstract

**Background:**

The secretory pathway is a critical index of the capacity of cells to incorporate proteins into cellular membranes and secrete proteins into the extracellular space. Importantly it is disrupted in response to stress to the endoplasmic reticulum that can be induced by a variety of factors, including expression of mutant proteins and physiologic stress. Activation of the ER stress response is critical in the etiology of a number of diseases, such as diabetes and neurodegeneration, as well as cancer. We have developed a highly sensitive assay to monitor processing of proteins through the secretory pathway and endoplasmic reticulum (ER) stress in real-time based on the naturally secreted Gaussia luciferase (Gluc).

**Methodology/Principle Findings:**

An expression cassette for Gluc was delivered to cells, and its secretion was monitored by measuring luciferase activity in the conditioned medium. Gluc secretion was decreased down to 90% when these cells were treated with drugs that interfere with the secretory pathway at different steps. Fusing Gluc to a fluorescent protein allowed quantitation and visualization of the secretory pathway in real-time. Expression of this reporter protein did not itself elicit an ER stress response in cells; however, Gluc proved very sensitive at sensing this type of stress, which is associated with a temporary decrease in processing of proteins through the secretory pathway. The Gluc secretion assay was over 20,000-fold more sensitive as compared to the secreted alkaline phosphatase (SEAP), a well established assay for monitoring of protein processing and ER stress in mammalian cells.

**Conclusions/Significance:**

The Gluc assay provides a fast, quantitative and sensitive technique to monitor the secretory pathway and ER stress and its compatibility with high throughput screening will allow discovery of drugs for treatment of conditions in which the ER stress is generally induced.

## Introduction

The endoplasmic reticulum (ER) is the intracellular organelle where proteins with a signal sequence are originally directed to be folded and glycosylated before they are processed through the secretory pathway destined for cell membranes, organelles or the extracellular space [1], [2]. Proteins enter the secretory pathway through translocons in the ER membrane in association with ER lumenal chaperones, such as calnexin, BiP and protein disulfide isomerase (PDI) [3]. Only properly folded proteins leave the ER within vesicles to the Golgi and misfolded proteins are transported back into the cytosol for degradation by proteosomes [4]. The ER lumen has a remarkable ability to maintain homeostasis and any physiological or pathological stimuli that leads to an increase in misfolded proteins, such as alterations in re-dox balance and calcium concentrations, glucose deprivation, presence of mutant proteins or even increased production of normal secretory proteins can trigger the ER stress response [5]. Activation of the ER stress response is critical in the etiology of a number of diseases, including diabetes and neurodegeneration, as well as cancer [6], [7]. Cells react to ER stress by activating a series of sensors termed the unfolded protein response (UPR), which leads to a temporary inhibition of protein synthesis and an increase in synthesis of ER chaperone proteins which promote protein folding, secretion and degradation to reduce the unfolded protein load in the ER [7].

Trafficking through the secretory pathway has traditionally been measured in the medium by using radioactively labeled endogenous glycoproteins [8], or by DNA transfection of cells with viral glycoproteins [9] or secreted alkaline phosphatase (SEAP) [10]. Visualization of the movement of proteins in the secretory pathway has been achieved using the thermoreversible folding mutant ts045 vesicular stomatitis virus G protein (VSVG) fused to enhanced green fluorescent protein (GFP) [11].

Blocking or decrease of processing in the secretory pathway is a hallmark of ER stress [5]. Many biological markers have been used to monitor ER stress in culture and/or in mice including: upregulation of mRNA or protein for the ER molecular chaperone, BiP [12]; PCR-based assays that detect stress induced mRNA splicing of the XBP-1 transcription factor [13]; and phosphorylation of PERK, eIF2alpha, ATF-4 and CHOP [14]. Other assays to monitor ER stress include: placement of a reporter, such as LacZ [15], GFP [16] or luciferase [12] under the control of an ER stress response element (ERSE); spliced activation of an XBP-1-venus fusion protein [16]; and changes in rates of SEAP secretion [17].

In this study, we describe a simple, highly sensitive assay for monitoring both the secretory pathway and ER stress in living mammalian cells based on expression of the naturally secreted Gluc [18] and monitoring release of luciferase activity in real-time. Parameters of Gluc secretion were monitored in cultured cells including linearity of release with time and cell number and response to drugs that either interfere with the secretory pathway or induce ER stress. Expression of Gluc in mammalian cells did not itself elicit an ER stress response, but induction of ER stress led to a temporary decrease in Gluc secretion which correlated with an increase in XBP-1 message splicing and levels of phosphorylated eIF2alpha, known ER stress indicators. Also, a fusion protein including Gluc and yellow fluorescence protein (YFP) allowed visualization of the secretory pathway within cells, as well as serving as a means of monitoring secretion of luciferase activity. The Gluc assay proved to be over 20,000-fold more sensitive in monitoring the secretory pathway in mammalian cells as compared to the SEAP assay in a range covering over 5 orders of magnitude with respect to cell number.

## Results

### Gluc as a reporter in mammalian cells

**Figure 1:**
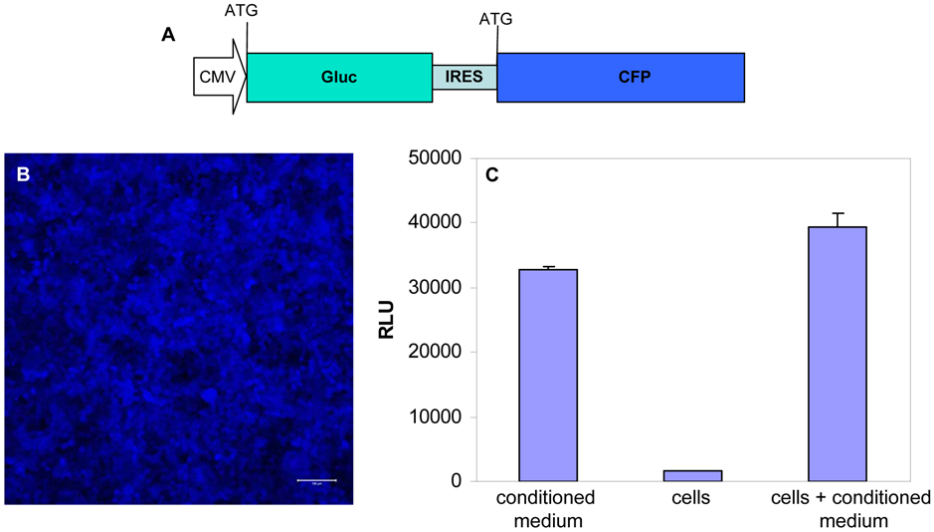
Gluc as a reporter in mammalian cells. (A) Schematic representation of the expression cassettes for Gluc-IRES-CFP cloned in the CSCW lentivirus vector. (B) High infection rate of cells with lentivirus vectors (M.O.I. = 30) as monitored by cerulean fluorescence. Scale bar, 100 µm. (C) Levels of Gluc activity in cells vs. medium vs. cells+medium. 293T cells were infected with the lentivirus vector carrying the expression cassette for Gluc-IRES-CFP and 20,000 cells were plated in wells of a 96-well plate. 48 hrs post-infection, new medium was added to the wells and Gluc activity was measured 24 hrs later in conditioned medium, viable washed cells or cells+conditioned medium after adding 2.5 µM coelenterazine.

A lentivirus vector was generated carrying the expression cassette for humanized Gluc [18] and the blue fluorescent protein, cerulean [19] separated by an internal ribosomal entry site (IRES) (**Fig. 1A**) and used to deliver Gluc and cerulean to >95% of human fibroblasts (293T cells) in culture by infection (**Fig. 1B**). In order to assess the level of secreted Gluc with respect to the intracellular level, 293T cells were infected with this lentivirus vector and 24 hrs later, Gluc activity was assayed in viable cells, the conditioned medium, or both. We observed that >95% of the expressed Gluc is secreted and thus assaying a combination of both intracellular and secreted levels corresponds mostly to the secreted level (**Fig. 1C**).

### Linearity and sensitivity of Gluc assay

**Figure 2:**
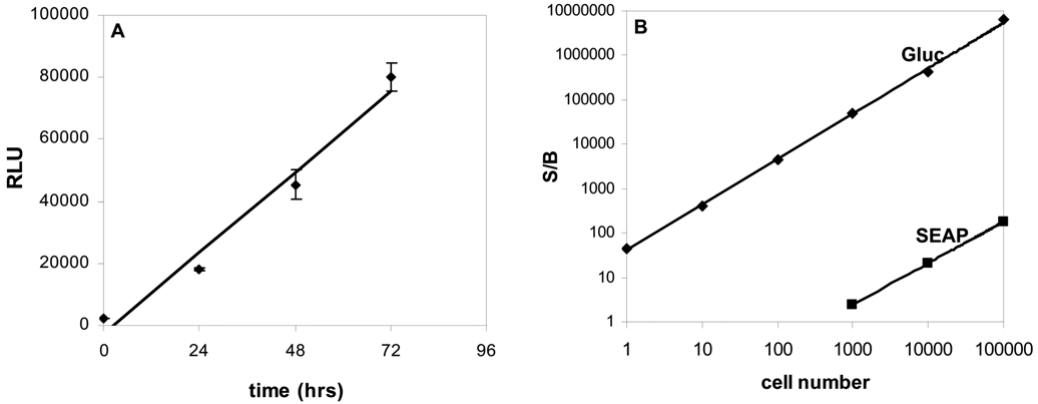
Linearity and sensitivity of the Gluc assay. (A) 293T cells were infected with the lentivirus vector carrying the expression cassette for Gluc-IRES-CFP and 20,000 cells were plated in wells of 96-well plate. Gluc activity was monitored over time in 10 µL of conditioned medium after addition of 20 µM coelenterazine. The release of Gluc to the conditioned medium is linear with time. (B) 293T cells were co-transfected with pHGC-Fluc and either pHGC-Gluc or pSEAP expression plasmids. Gluc and SEAP activities were assayed in conditioned medium 24 hrs later. The signal over background ratio (S/B) values, normalized to the intracellular levels of Fluc, are plotted against the number of cells. The Gluc assay can detect a single cell with S/B of 40 whereas the SEAP assay requires 20,000 cells to get similar S/B under similar assay conditions. The mean ± S.E.M. is presented on the graphs (n = 3).

In order to assess the linearity of Gluc with respect to time, 293T cells were infected with the lentivirus vector carrying the expression cassette for Gluc. Forty-eight hrs post-infection, fresh medium was added and the level of secreted Gluc was assayed overtime by taking an aliquot of the conditioned medium, adding coelenterazine, and measuring photon counts using a luminometer. The secreted bioluminescent signal of Gluc from cells into the conditioned medium was linear with respect to time over 72 hrs postinfection (**Fig. 2A**).

To compare the linearity of Gluc reporter with respect to cell number and its sensitivity compared to SEAP, a well established assay for monitoring the secretory pathway and ER stress [10], [17], [20], 293T cells were co-transfected with a plasmid carrying the expression cassette for firefly luciferase (Fluc; for normalization of transfection efficiency) and a plasmid carrying the expression cassette for either Gluc or SEAP. Different numbers of transfected cells ranging from a single cell to one hundred thousand cells were then plated in 96 well plates. Twenty-four hrs later, luminescent activity of Gluc (measured using coelenterazine) and SEAP (CSPD substrate) was measured in the cell-free conditioned medium, normalizing to the number of transfected cells by Fluc activity (_D_-luciferin) in cells. Under parallel assay conditions, as low as a single cell could be detected with a signal-to-background ratio (S/B) of 40 with the Gluc assay whereas 20,000 cells were required to get a similar S/B with the SEAP assay (**Fig. 2B**).

### *Gaussia* luciferase as a reporter for monitoring the secretory pathway

**Figure 3:**
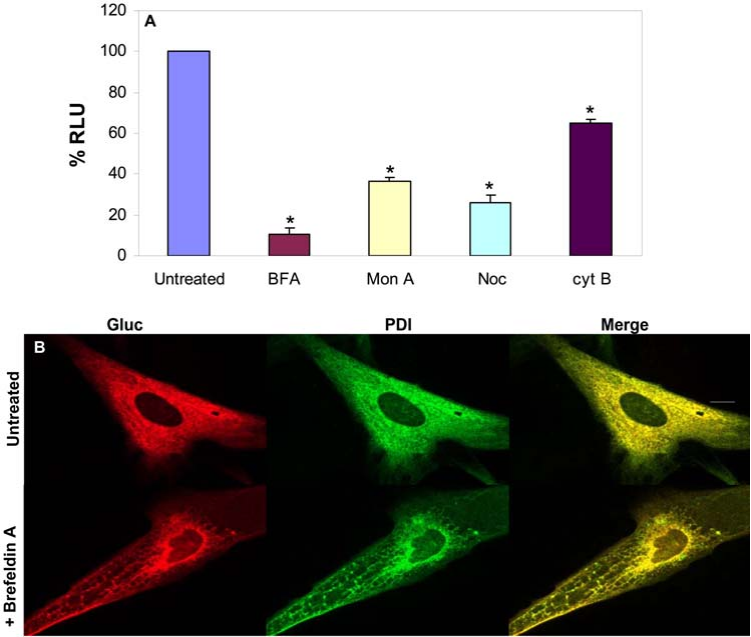
Gluc as a reporter to monitor secretory pathway. 293T cells were infected with the lentivirus vector expressing Gluc and were plated in wells of 96-well plate. Cells were treated for 24 hrs with different drugs which interfere with the secretory pathway. (A) Cell-free conditioned media were assayed for Gluc activity which showed that Gluc secretion is decreased upon blocking the secretory pathway. *p≤0.01 as predicted by student T-test. (B) Immunocytochemistry on cells expressing Gluc with and without treatment with BFA showing that Gluc (cy3, red) co-localizes with the ER marker PDI (Alexa488, green). Scale bar, 10 µm.

To determine whether Gluc is released via the conventional cell secretory pathway, i.e. rough ER, Golgi and vesicles, we inhibited secretory transport at different steps. 293T cells were treated with the following drugs: brefeldinA (BFA) which inhibits anterograde ER export to the Golgi, but allows retrograde Golgi-ER transport, resulting in a fusion of the ER and the Golgi and blocking of secretion [21]; monensin A which blocks transport within the Golgi [22]; nocodazole which causes microtubule depolymerization leading to blocking of microtubule-dependent translocation of vesicles between the ER and the Golgi [23]; and cytochalasin B which disrupts the actin cytoskeleton and thereby blocks membrane transport along actin filaments [24]. Treating cells with these drugs resulted in significant inhibition of Gluc secretion into the conditioned medium with about 90% decrease with BFA, 65% with monensin A, 75% with nocodazole, and 30% with cytochalasin B at the concentration used over 24 hrs (**Fig. 3A**). To investigate the location of Gluc within cells with and without treatment with BFA, HF8 human fibroblast cells expressing Gluc and the same cells treated with BFA were fixed with paraformaldehyde and co-stained with antibodies against Gluc and PDI, an ER marker. In both cases Gluc co-localized with PDI in the ER (**Fig. 3B**).

### Gluc-YFP fusion to quantitate and visualize secretory pathway in real-time

**Figure 4:**
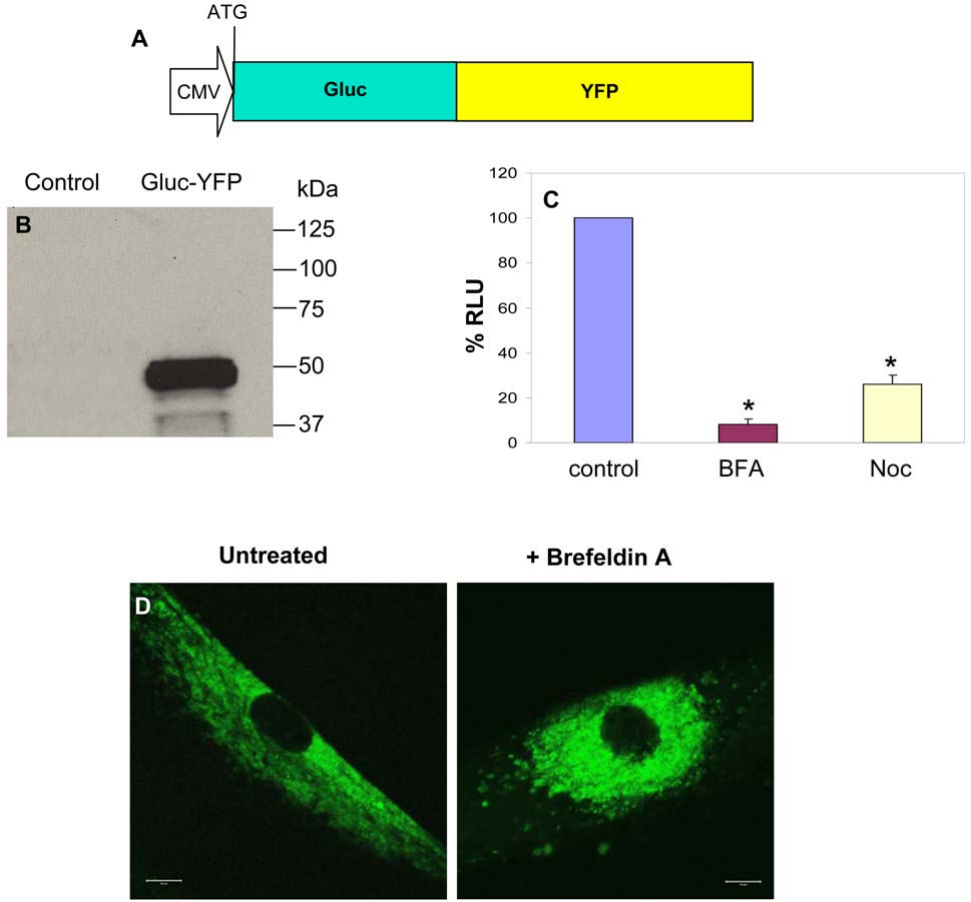
Gluc-YFP fusion to visualize secretory pathway in real-time. (A) Schematic representation of the Gluc-YFP fusion cloned in the lentivirus vector. (B) Uninfected 293T cells or cells infected with a lentivirus vector expressing Gluc-YFP fusion were lysed and analyzed by western blotting with anti-Gluc antibody. (C) Cells expressing Gluc-YFP fusion were treated with BFA or nocodazole and their conditioned medium were assayed for Gluc activity 24 hrs later. *p≤0.01 as predicted by student T-test. (D) Fluorescence microscopy of a single live cell expressing Gluc-YFP and either untreated or treated with BFA showing that this fusion is trapped in the ER upon BFA treatment. Scale bar, 10 µm.

In order to visualize movement of Gluc fate through the secretory pathway in real-time, we created a fusion protein with Gluc at the N-terminus and YFP at the C-terminus (**Fig. 4A**). HF8 cells were infected with a lentivirus vector carrying the expression cassette for Gluc-YFP and 48 hrs later lysates were analyzed by western blotting with anti-Gluc antibody revealing a band at 47 kDa, the predicted size for the fusion protein (**Fig. 4B**). This same band was also detected with an antibody against GFP (data not shown). Further, when the same cells were treated with BFA and nocodazole, secretion of Gluc-YFP luciferase activity was blocked to the same extent as for Gluc (**Fig. 4C**) and the fusion protein was also trapped in the ER upon BFA treatment as visualized by real-time confocal microscopy on live cells (**Fig. 4D**).

### *Gaussia* luciferase for monitoring of ER stress in real-time

**Figure 5:**
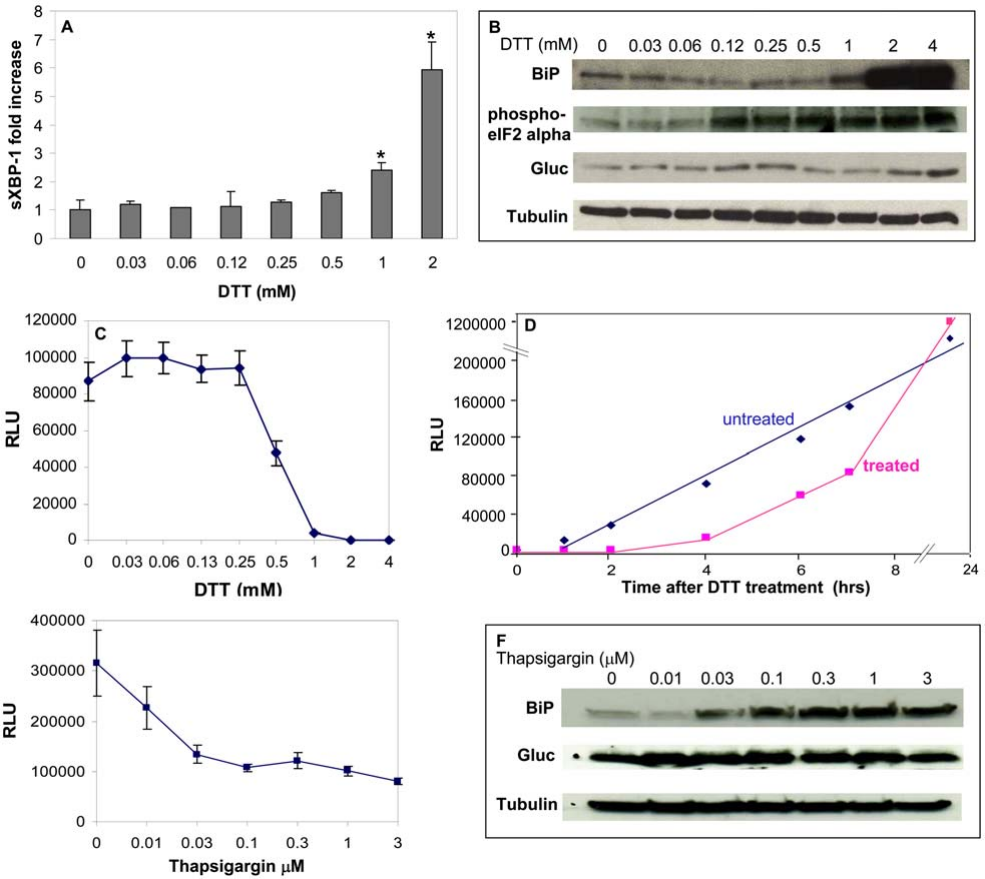
Monitoring of ER stress with Gluc. 293T cells expressing Gluc were subjected to no or different concentrations of DTT (A–D) or thapsigargin (E–F) to induce ER stress. (A) Real-time RT-PCR for spliced XBP-1 mRNA which increased in response to >1 mM DTT 4 hrs after treatment. Fold induction is calculated with respect to the control non-infected/non-treated cells. (B&F) Western blot analysis showing upregulation of BiP and/or phosphorylated eIF2alpha levels in response to ER stress 24 hrs after treatment. Blot is also probed with Gluc antibody as well as beta-tubulin antibody for equal loading. (C&E) Conditioned medium assayed for Gluc activity 4 hrs after DTT or thapsigargin treatment showing that Gluc secretion is decreased in response to ER stress. (D) Untreated or treated cells with 1 mM DTT were monitored overtime for the level of Gluc secretion by assaying an aliquot of the conditioned medium for Gluc bioluminescence. The mean ± S.E.M. is presented on the graphs (n = 3), with *p≤0.01 as calculated by the student's t-Test.

Splicing of the XBP-1 mRNA to sXBP1, expression of BiP protein and phosphorylated eIF2alpha are generally used as biomarkers for ER stress [5], [7]. In the assay developed here, infection of cells with the lentivirus vector and expression of the Gluc reporter did not induce ER stress in and of itself as evaluated by XBP-1 message splicing (**Fig 5A**), BiP levels and phosphorylated eIF2alpha (**Fig. 5B**), nor did it effect cell viability as assessed by tetrazolium salt, WST-1 cell proliferation assay (Roche Diagnostics GmbH, Mannheim, Germany) evaluated 48 hrs after infection of >95% of cells (data not shown). In order to investigate whether changes in Gluc secretion could be used as a marker for ER stress, 293T cells expressing Gluc were treated with different concentration of dithiothreitol (DTT; 0.03–4 mM) which induces ER stress [25]. Then three assays were carried out in parallel: 1) the cellular RNA was subjected to qRT-PCR for sXBP-1 (4 hrs exposure to DTT); 2) the cell lysates were resolved on SDS-PAGE followed by western blotting for BiP, phosphorylated eIF2alpha, Gluc and beta-tubulin (24 hrs); and 3) the conditioned medium was assayed for Gluc activity (4 hrs). Increasing the concentration of DTT increased the extent of XBP-1 mRNA splicing (**Fig. 5A**) and the amount of phosphorylated eIF2alpha (**Fig. 5B**) in a dose dependent manner, which inversely correlated with an early decrease in secretion of Gluc (**Fig. 5C**). Further, real-time monitoring of Gluc secretion in the conditioned medium upon 1 mM DTT treatment showed a complete inhibition of secretion for the first 3 hrs which started to recover after 4 hrs, and returned to normal level after 24 hrs (**Fig. 5D**) as the amount of BiP increases (**Fig. 5C**). To corroborate these results with a different ER-stress inducer, similar experiments were performed in which cells were incubated with different concentration of thapsigargin (0–3 µM) [25]. As expected, increasing concentration of this drug decreased the secretion of luciferase temporary in a similar way to DTT treatment and correlated with an increase in BiP expression (**Fig. 5E&F**). When tunicamycin (3 µg/ml) was used to induce ER stress [**25**], similar results were obtained with a 9-fold increase in spliced XBP-1 message and over 90% decrease in Gluc secretion (data not shown). These findings show that the Gluc reporter is a sensitive marker in elucidating ER stress in mammalian cells in real-time.

## Discussion

The facility of Gluc in reporting on the secretory pathway in mammalian cells by assaying their conditioned medium makes it a useful tool in monitoring processing of proteins and detecting ER stress. The Gluc assay described here is highly sensitive, quantitative and linearly related with respect to cell number in a range covering over five orders of magnitudes.

Use of Gluc has clear advantages as compared to other systems used to measure the secretory pathway and ER stress. The SEAP assay generally used to monitor ER stress [10], [17], [20] is also quantitative, but it is much less sensitive than the Gluc assay, is more time consuming to measure levels of activity, and requires sample dilution and different incubation times at different temperature before processing. Further, unlike the SEAP assay, the Gluc-YFP version of this reporter system allows the visualization of the secretory pathway in cells.

A well established assay for visualizing ER to Golgi transport is a fusion between VSVG and GFP by switching the temperature from 40°C at which the fusion is retained in the ER to 32°C at which the fusion is transported from the ER to the Golgi [11]. This reporter has the advantage that its passage through the secretory pathway can be arrested by a temperature shift and viewed at the intracellular level, but it does not allow quantitative assessment of secretion. In contrast, Gluc-YFP fusion allows visualization and quantitation but cannot be arrested in passage by a temperature shift, although it can be with drugs that block the secretory pathway.

Recently, another naturally secreted luciferase from the marine copepod *Metridia longa* has been characterized [26] and used as a marker for protein processing and ER stress. However, *Metridia* luciferase was shown to be less sensitive than the SEAP assay [17], [20] and the SEAP assay is much less sensitive (over 20,000-fold) than the Gluc assay described here. The naturally secreted Gluc reporter provides a facile, sensitive assay for monitoring the secretory pathway and ER stress and the expression cassette for it can be delivered to cells by either transfection and infection, with the latter expanding the number of cell types that can be tested and allowing for stable expression in progeny cells. Further, this assay is compatible with high throughput drug screening since all that is required for measurement is coelenterazine, its substrate, and a plate luminometer which measures its reaction in few seconds and there is no need to remove the conditioned medium (since >95% of Gluc is secreted) if one prefers to assay in the same well where cells are plated and not overtime.

The Gluc secretion assay provides a highly sensitive, facile way to monitor processing of proteins through the secretory pathway. Thus it can be used to dissect out components of this pathway, for example in elucidating the action of mutant torsinA responsible for early onset torsion dystonia in interfering with protein secretion [27] and in monitoring ER stress, which is implicated in a number of diseases, for example diabetes and neurodegeneration [7]. This assay will provide a means to identify drugs which can counteract the effects of mutant proteins using high throughput screens, and has the potential to monitor effects of these drugs on ER stress in vivo.

## Methods

### Cell culture

293T human embryonic kidney fibroblasts were obtained from Dr. David Baltimore, Massachusetts Institute of Technology, Cambridge, MA, USA[28]. HF8 human fibroblast cells from a normal donor were generated in our laboratory[29]. Cells were cultured in Dulbecco's modified Eagle's medium (DMEM) supplemented with 10% fetal bovine serum (Sigma, St. Louis, MO), 100 U penicillin, and 0.1 mg streptomycin (Sigma) per milliliter, at 37°C in a 5% CO_2_ humidified incubator.

### Lentiviral construct

CSCW-IG is a self-inactivating lentiviral vector which has a CMV immediate early promoter controlling transgene and the GFP cDNA separated by an IRES element [30]. The cDNA encoding hGluc [18] and the optimized blue fluorescent protein, cerulean (CFP, from Dr. David Piston, Vanderbilt Univ. Med. Ctr., TN)[19] were amplified by PCR. Gluc was cloned directly downstream of the CMV promoter and the CFP was cloned in place of the GFP cDNA generating pCSCW-Gluc-IRES-CFP lentivirus vector construct (**Fig. 1A**). The Gluc-YFP fusion was generated by placing the YFP cDNA (Clontech) in-frame directly downstream of the coding sequences of Gluc cDNA lacking the stop codon. This fusion was cloned in the previous vector in place of Gluc-IRES-CFP generating CSCW-Gluc-YFP (**Fig. 1B**). Lentivirus vectors were prepared as described [30]. In brief, 293T cells were co-transfected with the pCSCW-Gluc-IRES-CFP or pCSCW-Gluc-YFP plasmids, the lentivirus packaging genome CMVRΔ8.91 (from Dr. Didier Trono, Univ. Geneva, Switzerland) and envelope coding plasmid (pVSV-G; provided by Dr. Miguel Sena-Esteves, MGH) [30]. After 72 hrs, lentivirus vector supernatant was harvested, concentrated by ultracentrifugation and titered as transducing units (t.u.)/ml on 293T cells in the presence of 10 µg/mL polybrene (Sigma) by counting the CFP or YFP positive cells 48 hrs post-infection. A typical titer is around 10^8^ t.u./ml.

### Sensitivity of Gluc versus SEAP

293T cells were plated in 60 mm dishes (5×10^5^ cells/dish) and co-transfected with a plasmid encoding Fluc under control of CMV promoter (pHGC-Fluc) [18] and either a plasmid encoding Gluc (pHGC-hGluc) [18] under the control of CMV promoter or a plasmid encoding secreted alkaline phosphatase (pSEAP2-control vector, Clontech) under control of SV40 early promoter using Lipofectamine (Invitrogen, Carlsbad, CA). Twenty-four hrs after transfection, cells were harvested, washed with PBS and different numbers of cells were plated in a well of 96 well plate. Twenty-four hrs later, cell-free conditioned medium were assayed for either Gluc or SEAP. Transfection efficiency was normalized to the level of Fluc activity in viable cells.

### Luciferase activity

Gluc activity was measured by adding 20 µM coelenterazine (Prolume Ltd./Nanolight, Pinetop, AZ) to an aliquot of the cell-free conditioned medium and measured for 10 sec using a luminometer (Dynex, Richfield, MN). For Fluc detection, 450 µM Beetle _D_-luciferin (Molecular Imaging Products, Ann Arbor, MI) was added directly to the viable cells, in a 96 well plate and measured as above. The signal was measured for 10 sec and integrated over 2 sec in both cases.

### Secreted alkaline phosphatase (SEAP) assay

An aliquot of the conditioned cell-free medium was used to monitor the SEAP activity using the Great EscAPe SEAP kit (Clontech). Briefly, 15 µL cell free medium was mixed with 45 µL of 1× dilution buffer and incubated at 65°C for 30 min. Samples were cooled down to room temperature and mixed with 60 µL assay buffer, incubated for 5 min at room temperature before adding 60 µL of chemiluminescent enhancer containing 1.25 mM CSPD substrate (3-(4-methoxyspiro{1,2-dioxetane-3,2′-(5′-chloro)-tricyclo[3.3.1.1.3,7]-decan}-4-yl) phenyl phosphate). After 15 min of incubation at room temperature, chemiluminescence was measured using a luminometer.

### ER stress: total RNA isolation, reverse transcription and real-time qPCR

293T cells were plated at 1×10^6^ cells/well in a 12-well plate. Cells were treated with different concentrations of DTT (Sigma) or thapsigargin (Sigma) for 4 hrs. Total RNA was isolated using the RNeasy mini kit (Quiagen, Valencia, CA) and concentration was determined by measuring the OD_260_ using a spectrophotometer (Bio-Rad, Hercules, CA). Reverse transcription was performed on 200 ng RNA (in 10 µl) at 37°C for 60 min using an Omniscript reverse transcription kit (Quiagen). Two µL of each cDNA sample were used for real-time PCR. Biological triplicates were measured twice. The following primers were used: Forward 5′-GGTCTGCTGAGTCCGCAGCAGG-3′ and Reverse 5′-GGGCTTGGTATATATGTGG-3′. These primers were designed to span a 26 bp intron in the unspliced XBP1 mRNA [13]. For normalization, we used human GAPDH primers: Forward 5′-TGGAAAGCTGTGGCGTGATGGCCG-3′ and Reverse 5′-CACCCAGAAGACTGTGGATGGCCCCT-3′. Real-time PCR was performed in an ABI PRISM 7000 Sequence Detection System Thermal Cycler (Applied Biosystems, Foster City, CA) in a total volume of 30 µL, using the SYBR green PCR master mix (Applied Biosystems) with 10 pico-moles of the primers set. The fold increase was calculated based on Ct values of treated cells relative to control non-infected and non-treated cells normalized to the values for GAPDH endogenous control.

For real-time monitoring of ER-stress with Gluc, 293T cells infected with lentivirus vector carrying the expression cassette for Gluc. Forty-eight hrs later, cells were treated with different concentration of DTT and aliquots of the conditioned medium were assayed for Gluc activity either 4 hrs later (For the DTT and thapsigargin dose-response curve) or at different time points (for real-time monitoring of ER-stress) as above.

### Western blot

Twenty-four hrs after DTT or thapsigargin treatment, total cell lysates were prepared in lysis buffer containing 150 mM NaCl, 50 mM TRIS, pH 8.0, 1% NP-40, 0.5% deoxycholate, 0.1% SDS, and protease inhibitors (PI Complete; Boehringer Mannheim, Indianapolis, IN). Forty µg protein were electrophoresed in 12.5% SDS-polyacrylamide gels, and transferred to nitrocellulose membranes (Bio-Rad). Membranes were blocked overnight in 10% non-fat milk powder in TBST (150 mm NaCl, 50 mm TRIS, pH 7.9, 0.5% TWEEN) and probed with antibodies against Gluc (1∶500; prepared by the Neuroscience Center Monoclonal Antibody Production Core at Mass. General Hospital), BiP (1∶200; Stressgene, College, PA), phosphorylated eIF2alpha (1∶1000; Cell signaling, Danvers, MA) or β-tubulin (Sigma) diluted in TBST. Membranes were then incubated with horseradish peroxidase (HRP) conjugated to secondary antibodies: sheep anti-mouse IgG-HRP or donkey anti-rabbit IgG-HRP (1∶10,000; Amersham Pharmacia Biotech, Piscataway, NJ). For protein detection we used SuperSignal West Pico Chemiluminescent Substrate™ (Pierce, Rockford, IL).

### Blocking of secretory pathway

293T cells were infected with the lentivirus vector expressing Gluc. Forty-eight hrs post-infection, cells were treated with either 5 µg/ml BFA, 3 µg/ml monensin A (MonA), 10 µg/ml nocodazole (Noc) or 5 µg/ml cytochalasin B (CytB) all obtained from Sigma. Twenty-four hrs later, the Gluc activity was measured in the conditioned medium as above.

### Immunocytochemistry

HF8 human fibroblast cells were plated on coverslips (100 cells/coverslip) and control or BFA treated cells (for 24 hrs) were extracted with digitonin. Cells were then fixed with 4% paraformaldehyde in PBS for 10 min at room temperature, washed with PBS and incubated with 0.1% NP-40 in PBS for 10 min. Blocking was performed using 10% goat serum (Vector Laboratories, Burlingame, CA) in PBS for 1 hr. Cells were incubated with monoclonal mouse anti-Gluc antibody (1∶100) and PDI (Stressgene, 1∶600), for 1 hr at 37°C. For fluorescence detection we used secondary antibodies, conjugated to Cy3 affiniPure donkey anti-mouse (1∶1000; Jackson Immuno Labs, West Grove, PA) or Alexa 488 goat anti-rabbit (1∶2000; Invitrogen-Molecular Probes, Carlsbad, CA). Coverslips were mounted onto slides using gelvatol mounting medium containing 15 µg/ml anti-fade agent 1,4-diazabicyclo(2.2.2)-octane (Sigma). Images were captured using an inverted fluorescent microscope (Nikon TE 200-U) coupled to a digital camera.

## References

[1] Ellgaard L, Helenius A (2003). Quality control in the endoplasmic reticulum.. Nat Rev Mol Cell Biol.

[2] Voeltz GK, Rolls MM, Rapoport TA (2002). Structural organization of the endoplasmic reticulum.. EMBO Rep.

[3] Stevens FJ, Argon Y (1999). Pathogenic light chains and the B-cell repertoire.. Immunol Today.

[4] Tsai B, Ye Y, Rapoport TA (2002). Retro-translocation of proteins from the endoplasmic reticulum into the cytosol.. Nat Rev Mol Cell Biol.

[5] Zhang K, Kaufman RJ (2006). The unfolded protein response: a stress signaling pathway critical for health and disease.. Neurology.

[6] Ozcan U, Cao Q, Yilmaz E, Lee AH, Iwakoshi NN (2004). Endoplasmic reticulum stress links obesity, insulin action, and type 2 diabetes.. Science.

[7] Zhao L, Ackerman SL (2006). Endoplasmic reticulum stress in health and disease.. Curr Opin Cell Biol.

[8] Bertrand F, Veissiere D, Hermelin B, Paul A, Capeau J (1994). Phosphorylation of vimentin is an intermediate step in protein kinase C-mediated glycoconjugate secretion.. Am J Physiol.

[9] de Silva AM, Balch WE, Helenius A (1990). Quality control in the endoplasmic reticulum: folding and misfolding of vesicular stomatitis virus G protein in cells and in vitro.. J Cell Biol.

[10] Berger J, Hauber J, Hauber R, Geiger R, Cullen BR (1988). Secreted placental alkaline phosphatase: a powerful new quantitative indicator of gene expression in eukaryotic cells.. Gene.

[11] Presley JF, Cole NB, Schroer TA, Hirschberg K, Zaal KJ (1997). ER-to-Golgi transport visualized in living cells.. Nature.

[12] Lee YS, Kim HK, Chung S, Kim KS, Dutta A (2005). Depletion of human micro-RNA miR-125b reveals that it is critical for the proliferation of differentiated cells but not for the down-regulation of putative targets during differentiation.. J Biol Chem.

[13] Hirota M, Kitagaki M, Itagaki H, Aiba S (2006). Quantitative measurement of spliced XBP1 mRNA as an indicator of endoplasmic reticulum stress.. J Toxicol Sci.

[14] Li F, Hayashi T, Jin G, Deguchi K, Nagotani S (2005). The protective effect of dantrolene on ischemic neuronal cell death is associated with reduced expression of endoplasmic reticulum stress markers.. Brain Res.

[15] Mao C, Dong D, Little E, Luo S, Lee AS (2004). Transgenic mouse model for monitoring endoplasmic reticulum stress in vivo.. Nat Med.

[16] Iwawaki T, Akai R, Kohno K, Miura M (2004). A transgenic mouse model for monitoring endoplasmic reticulum stress.. Nat Med.

[17] Hiramatsu N, Kasai A, Hayakawa K, Yao J, Kitamura M (2006). Real-time detection and continuous monitoring of ER stress in vitro and in vivo by ES-TRAP: evidence for systemic, transient ER stress during endotoxemia.. Nucleic Acids Res.

[18] Tannous BA, Kim DE, Fernandez JL, Weissleder R, Breakefield XO (2005). Codon-optimized Gaussia luciferase cDNA for mammalian gene expression in culture and in vivo.. Mol Ther.

[19] Rizzo MA, Springer GH, Granada B, Piston DW (2004). An improved cyan fluorescent protein variant useful for FRET.. Nat Biotechnol.

[20] Hiramatsu N, Kasai A, Meng Y, Hayakawa K, Yao J (2005). Alkaline phosphatase vs luciferase as secreted reporter molecules in vivo.. Anal Biochem.

[21] Lippincott-Schwartz J, Yuan LC, Bonifacino JS, Klausner RD (1989). Rapid redistribution of Golgi proteins into the ER in cells treated with brefeldin A: evidence for membrane cycling from Golgi to ER.. Cell.

[22] Tartakoff A, Vassalli P, Detraz M (1978). Comparative studies of intracellular transport of secretory proteins.. J Cell Biol.

[23] Rogalski AA, Singer SJ (1984). Associations of elements of the Golgi apparatus with microtubules.. J Cell Biol.

[24] Forer A, Emmersen J, Behnke O (1972). Cytochalasin B: does it affect actin-like filaments?. Science.

[25] Shang J (2005). Quantitative measurement of events in the mammalian unfolded protein response.. Methods.

[26] Markova SV, Golz S, Frank LA, Kalthof B, Vysotski ES (2004). Cloning and expression of cDNA for a luciferase from the marine copepod Metridia longa. A novel secreted bioluminescent reporter enzyme.. J Biol Chem.

[27] Hewett JW, Tannous BA, Niland BB, Nery FC, Zeng J, Li Y, Breakefield OX (in press). Mutant torsinA interferes with protein processing through the secretory pathwat in DYT1 dystonia cells.. Proc Natl Acad Sci USA.

[28] Pear WS, Nolan GP, Scott ML, Baltimore D (1993). Production of high-titer helper-free retroviruses by transient transfection.. Proc Natl Acad Sci U S A.

[29] Breakefield XO, Braverman M, Riker DK, Giller EL (1981). Catechol-O-methyltransferase activity in cultured human skin fibroblasts from controls and patients with dystonia musculorum deformans.. J Neurosci Res.

[30] Sena-Esteves M, Tebbets JC, Steffens S, Crombleholme T, Flake AW (2004). Optimized large-scale production of high titer lentivirus vector pseudotypes.. J Virol Methods.

